# Development of a SNP array and its application to genetic mapping and diversity assessment in pepper (*Capsicum* spp.)

**DOI:** 10.1038/srep33293

**Published:** 2016-09-13

**Authors:** Jiaowen Cheng, Cheng Qin, Xin Tang, Huangkai Zhou, Yafei Hu, Zicheng Zhao, Junjie Cui, Bo Li, Zhiming Wu, Jiping Yu, Kailin Hu

**Affiliations:** 1 College of Horticulture, South China Agricultural University, Guangzhou 510642, China; 2 Pepper Institute, Zunyi Academy of Agricultural Sciences, Zunyi, Guizhou 563102, China; 3 Guizhou Provincial College-based Key Lab for Tumor Prevention and Treatment with Distinctive Medicines, Zunyi Medical University, Zunyi, Guizhou 563000, China; 4 Guangzhou Genedenovo Biotechnology Co., Ltd, Guangzhou 510006, China; 5 BGI-Shenzhen, Shenzhen 518083, China; 6 Department of Computer Science, City University of Hong Kong, Hong Kong 999077, China; 7 College of Horticulture and Landscape Architecture, Zhongkai University of Agriculture and Engineering, Guangzhou 510225, China

## Abstract

The development and application of single nucleotide polymorphisms (SNPs) is in its infancy for pepper. Here, a set of 15,000 SNPs were chosen from the resequencing data to develop an array for pepper with 12,720 loci being ultimately synthesized. Of these, 8,199 (~64.46%) SNPs were found to be scorable and covered ~81.18% of the whole genome. With this array, a high-density interspecific genetic map with 5,569 SNPs was constructed using 297 F_2_ individuals, and genetic diversity of a panel of 399 pepper elite/landrace lines was successfully characterized. Based on the genetic map, one major QTL, named *Up12.1*, was detected for the fruit orientation trait. A total of 65 protein-coding genes were predicted within this QTL region based on the current annotation of the Zunla-1 genome. In summary, the thousands of well-validated SNP markers, high-density genetic map and genetic diversity information will be useful for molecular genetics and innovative breeding in pepper. Furthermore, the mapping results lay foundation for isolating the genes underlying variation in fruit orientation of *Capsicum*.

Peppers (*Capsicum* spp.) are native to the tropics and subtropics of Americas^1^, and belong to the large Solanaceae family, which includes a variety of valuable crops such as tomato (*Solanum lycopersicum*) and potato (*Solanum tuberosum*). Archeological microfossil evidence indicated that peppers were domesticated in the Americas and have been consumed in this region for more than 5,000 years^2^,^3^. Now five economically important domesticated species, including *C. annuum*, *C. frutescens*, *C. chinense* Jacq., *C. baccatum* and *C. pubescens* Ruiz & Pavon, are cultivated globally as fresh vegetables, seasoning, ornaments and medicine^4^.

Because of the vital economic importance of pepper, tremendous efforts have been expended to improve pepper variety and great progress has been made over the past decades. The development and application of DNA markers represent particularly important milestones^4^. To date, various marker systems, including restriction fragment length polymorphisms (RFLPs), random amplified polymorphic DNA (RAPD), amplified fragment length polymorphisms (AFLPs) and simple sequence repeats (SSRs), have been developed and utilized to assess diversity^5^,^6^,^7^, construct genetic maps^8^,^9^,^10^ and perform marker assisted selection (MAS)^11^,^12^ in pepper. However, the majority of these are still first- or second-generation markers. In addition, the limitations of anonymous markers, non-specificity, low density and incomplete coverage pose challenges to applications of these markers in modern genetic studies of pepper, especially given its large genome^13^,^14^.

Until recently, third generation marker systems such as single nucleotide polymorphisms (SNPs)^5^,^15^,^16^,^17^ and insertion/deletion polymorphisms (InDels)^18^,^19^ were gradually be discovered in large quantities in pepper through the rapid development of next generation sequencing (NGS). SNPs in particular, which possess obvious advantages such as high density across the genome^20^ and ease of automatic genotyping^21^, have emerged as the main focus of genetic polymorphism studies in pepper^22^,^23^,^24^. Nevertheless, the validation of these *in silico* SNPs through laboratory experiments and practical breeding in pepper merits further study.

Over the course of domestication, several plant and yield traits in pepper were specifically selected for adaption to cultivation and human demands^25^. Of these, the transformation of fruit-tip orientation from wild-type erect to cultivated pendulous is particularly important. This change may be associated with an increase in fruit size or better protection from threats by biotic and abiotic factors^25^. As a unique plant attribute of pepper, the erect positioning of fruits is simply inherited and controlled by the *up* locus^26^,^27^, which has been localized to chromosome P12^28^,^29^. Furthermore, molecular markers linked to the *up* locus, including A2C7_469_ (1.7 cM) and *up*CAPS (4.3 cM), have also been developed^27^. However, accurate and consistent physical candidate regions have yet to be delimited due to the previous lack of reference sequence information. Recently, six QTLs responsible for variation in pepper fruit orientation were identified based on an intraspecific population^16^. Nevertheless, the key genes controlling the major QTLs have not been discovered, and the underlying molecular regulation of fruit orientation in pepper is largely unknown.

Thus, in the current study, we first describe the development of an Illumina Infinium iSelect SNP array for pepper. We then used this newly developed array to genotype 297 F_2_ progenies of a pepper interspecific (*C. annuum* × *C. frutescens*) population, together with a panel of 399 pepper elite/landrace lines. Based on the genotype data, a genetic map was constructed and genetic diversity of the pepper panel was characterized. We expected that the thousands of well-validated SNP markers, genetic map and diversity information reported here would be useful for downstream applications in both basic and applied researches in pepper. In addition, we performed QTL analysis for the fruit orientation and hoped the related information would lay a foundation for further isolation of genes underlying the *up* locus of pepper.

## Results

### Development of a SNP array for pepper

Genome-wide comparison allowed us to identify a total of 4,762,278 predicted SNPs between BA3 and B702 (data not shown). Based on the filtering criteria mentioned in the Methods, a final set of 15,000 SNPs was submitted to develop the Illumina Infinium iSelect SNP array, hereafter referred to as *Cap*SNP15K. A total of 12,720 bead types were ultimately synthesized. Of these, 8,199 (~64.46%) SNP loci were scored with normal signals among all 1,019 investigated samples (Table 1), and the detailed genomic coordinates, variation types and flanking sequences of these scorable SNP loci were given in Supplementary Table S1. The loci comprised four types of allele variations (Supplementary Figure S1A), the majority of which were located in the intergenic regions (Supplementary Figure S1B) based on the annotation of the Zunla-1 genome (release 2.0). More importantly, these scorable SNPs anchored a total of 5,107 scaffolds, which covered 2,719,081,414bp of physical length and accounted for approximately 81.18% of the Zunla-1 genome assembly (Table 1). In addition, preliminary tests using 6 DNA samples showed that the duplicate reproducibility and the P-P-C heritability of this array were 100% and greater than 90.00% (Supplementary Tables S2 and S3), respectively.

### Construction of a high-density interspecific SNP genetic map of pepper

**Figure 1:**
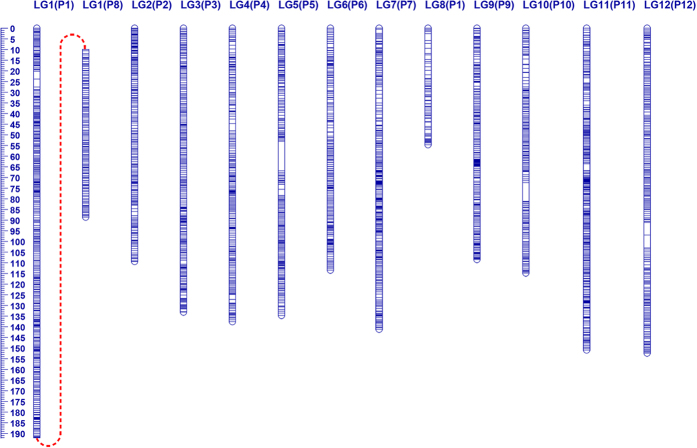
The high-density interspecific genetic map (BY-SNP) of pepper. This genetic map comprises 12 linkage groups (LG1-LG12) with 5,569 SNP markers. The corresponding chromosome numbers are shown in brackets, and the pseudo-linkage between P1 and P8 is shown with a dashed red line.

After the removal of 156 low quality loci from the 5,828 scorable SNPs within the mapping population, the retained 5,672 (97.32%) polymorphic SNPs were used for map construction. Finally, a high-density genetic map with 5,569 SNP markers forming 3,826 genetic bins was obtained and designated BY-SNP map (Fig. 1 and Table 2). Assigning the 12 linkage groups (LGs) of the BY-SNP map to the Zunla-1 genome revealed a high degree of consistency between the genetic and physical map of pepper (Supplementary Table S4). Meanwhile, a pseudo-linkage between the chromosomes P1 and P8 was observed (Supplementary Table S4). This might be due to reciprocal translocation or duplication between the *C. annuum* and *C. frutescens* genomes^18^,^30^, or distorted distorted markers being mapped in that region. What’s more, probably due to insufficient linkage, chromosome P1 was divided into two LGs, namely LG1 and LG8, with the breakpoint located on the upper end (44.97–77.11 Mb) of P1 (Fig. 1 and Supplementary Table S4). The average number of markers per LG was 318.83, with an average density of one bin marker for every 0.45 cM. Moreover, the total coverage of BY-SNP map was 1,628.83 cM and 2230.77 Mb for genetic and physical length, respectively.

**Figure 2:**
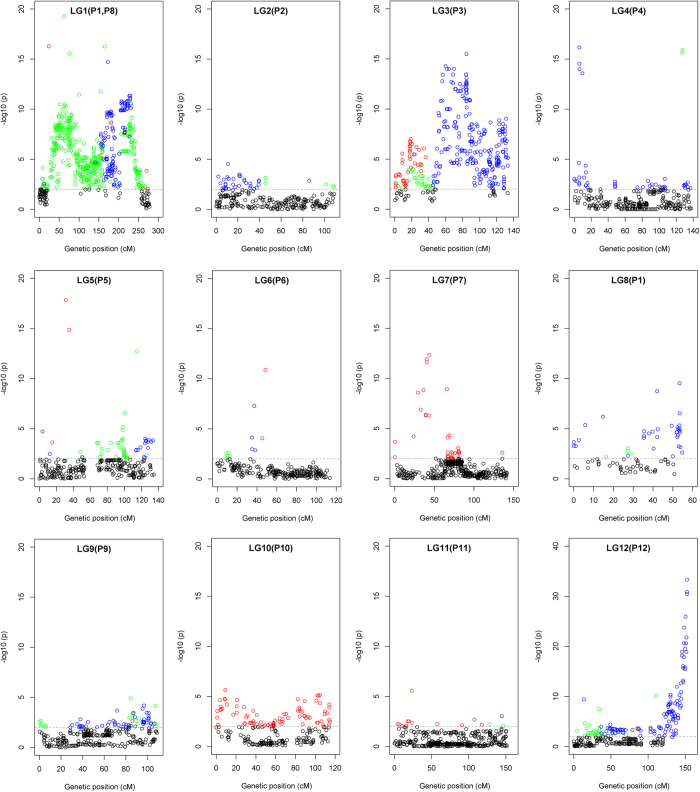
Extent and orientation of the distorted segregation of SNP markers in the BA3 × YNXML interspecific population. This analysis was performed on 4,879 SNP markers with a missing rate less than 0.1 among the 297 F_2_ progenies, and those with a chi-square test *P* value smaller than 0.01 were regarded as distorted markers. The skewed orientation towards BA3, YNXML and F_1_ is shown in red, blue and green, respectively, and 4 SNPs skewed to YNXML and F_1_ are shown in orange. All undistorted markers are presented in black.

Finally, as many as 1,672 of 4,879 (34.27%) SNPs showed distorted segregation (*P* < 0.01) in this interspecific population and 39 SDRs were identified on all chromosomes except for P6, P7 and P11 (Fig. 2 and Table 2). The skewed orientation seemed to be non-random. The majority of the skewed SNPs were towards YNXML (846 SNPs, blue colour) and the hybrid (619 SNPs, green colour), mainly distributing on chromosomes P1, P3, P8 and P12. Whereas all skewed markers on the chromosome P7 and P10 were towards BA3, which was consistent with the results inferred from InDel and SSR markers^18^. All this information should be useful for the identification of cryptic factors underlying segregation distortion in the future^31^.

### Genetic and QTL analysis of fruit orientation in pepper

In the present study, we found that all F_1_ individuals from BA3 and YNXML presented lateral pendant (LP) fruit orientation phenotype (Supplementary Figure S2B), which was distinct from the orientation of their parents (Supplementary Figure S2A,C). As for the 177 F_2_ individuals investigated, 50 showed an erect (E) fruit phenotype like YNXML (Supplementary Figure S2A), whereas 127 individuals exhibited a pendant (P) phenotype. However, the 127 pendant (P) plants could be further clearly divided into 97 lateral (LP) (Supplementary Figure S2B) and 30 vertical (VP) types (Supplementary Figure S2C). Chi-square tests indicated that the E: P and E: LP: VP ratios fit the expected ratio 3:1 (χ^2^ = 1.00, *P* = 0.61) and nearly matched the 1:2:1 expected ratio (χ^2^ = 6.15, *P* = 0.05), respectively. These results suggested that the erect fruit phenotype was under single recessive gene control and that the pendant fruit was controlled by a single dominant gene with incomplete inheritance.

**Figure 3:**
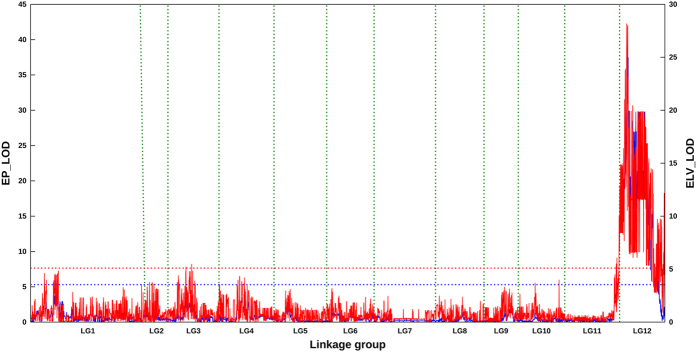
Likelihood profile of QTL mapping with phenotypic datasets from EP and ELV methods. Blue and red solid lines depict the EP and ELV methods, respectively. The genome-wide threshold LOD values for EP and ELV are shown as blue (LOD = 5.3) and red (LOD = 5.1) dashed lines, respectively.

Based on the BY-SNP framework map, one major QTL, named *Up12.1*, was consistently detected on LG12 (chromosome P12) regardless of whether the EP or ELV classification method was used (Fig. 3 and Table 3). This QTL explained more than 50% of the phenotypic variance. Furthermore, the 1-LOD-drop genetic and physical confidence intervals (CIs) were approximately 3.52 cM and 4.52 Mb, respectively (Table 3). A total of 65 protein-coding genes were predicted within this CI based on the current annotation of the Zunla-1 genome. Of which, 29 genes encode the known proteins such as 6-phosphofructokinase 1 (PFK1), Purine permease 3 (PUP3), Developmentally regulated G-protein 2 (DRG2) etc., whereas more than half are new genes (Supplementary Table S5).

### Genetic diversity and population structure among the panel of pepper lines

**Figure 4:**
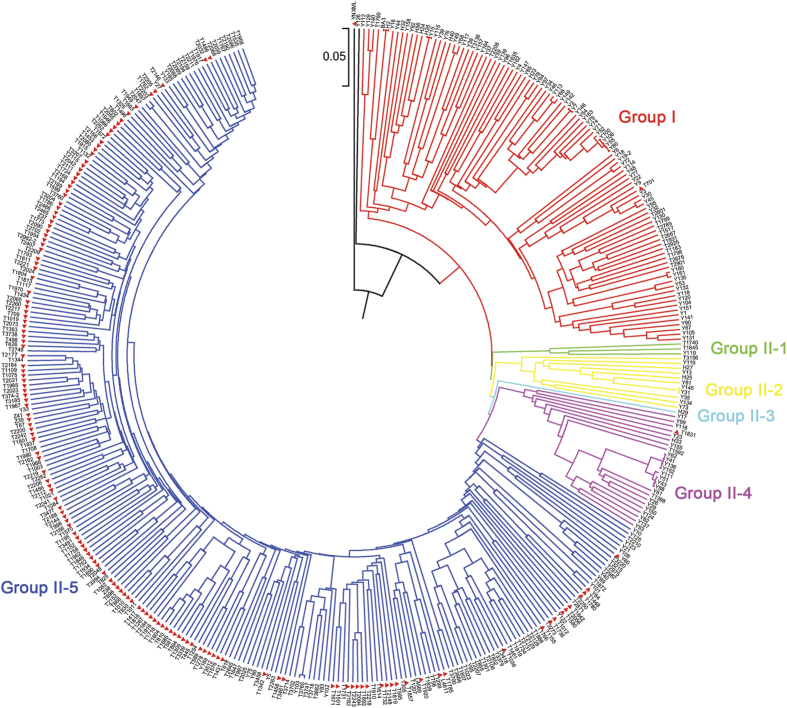
Phylogenetic tree of 399 pepper lines inferred from 5,149 SNP markers. The erect lines are labelled with solid red triangles.

A total of 5,149 filtered SNPs were used to evaluate the genetic diversity of the 399 pepper lines (Supplementary Tasble S6) and the summary statistics for this panel are presented in Table 4. The average gene diversity, heterozygosity and polymorphism information content (PIC) among the whole pepper panel were 0.36, 0.17 and 0.29, respectively. In addition, an UPGMA-phylogenetic tree constructed based on genetic distance showed that these SNP markers could classify the 399 lines into two major groups, including group I with 101 lines and group II with 296 lines, except for lines YNXML (outgroup) and Y126 (Fig. 4 and Table 4, Supplementary Table S6). Group II can be further divided into 5 sub-groups designated II-1 to II-5 (Fig. 4). Of these, the group II-5 consisted of 256 lines and showed the highest gene diversity of 0.33 (Table 4).

**Figure 5:**
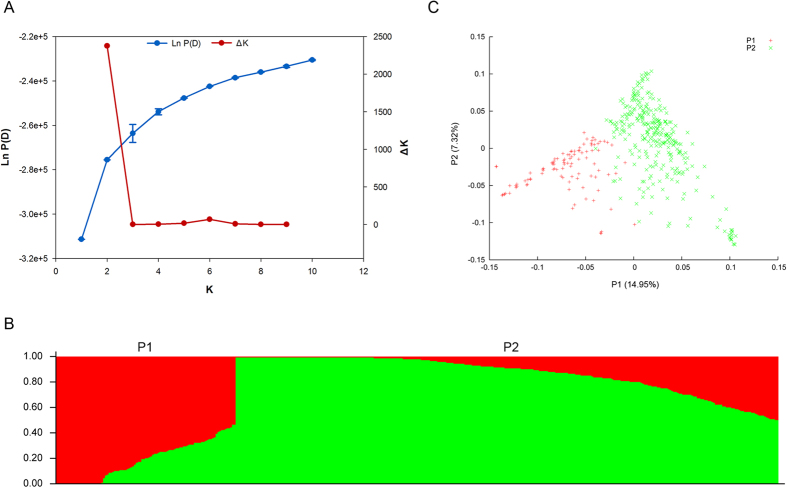
Representation of genetic structure of 398 *C. annuum* lines. (**A**) Estimated LnP (D) and ΔK values for different numbers of populations assumed (K, from 1 to 10) based on 833 SNPs; (**B**) Two population stratifications (P1 in red and P2 in green) based on the optimal K = 2; (**C**) PCA plot of the 398 *C. annuum* lines based on 6,470 SNPs (P1 in red and P2 in green).

On the other hand, as shown in Fig. 5A, the peak value of ΔK, for K = 2, suggested that the 398 *C. annuum* lines perhaps belonged to two distinct populations, which were designated P1 and P2, respectively (Fig. 5B). P1 and P2 consisted of 99 and 299 lines, respectively. The grouping results obtained via model-based population structure analysis were similar to that of clustering analysis based on genetic distance (Fig. 4). For example, 89 out of 99 (89.90%) lines from population P1 belonged to the group I, and 286 (96.62%) lines in group II (total is 296) were assigned to population P2 (Supplementary Table S7). In addition, the principal component analysis (PCA) displayed a similar clustering pattern of relationship to that of model-based population structure analysis (Fig. 5C).

## Discussion

Although SNPs have become the marker of choice for the scientific and breeding community^20^, the development and application of pepper SNPs remain in their infancy^5^,^15^ but are becoming increasingly important. In particular, since the start of the pepper (Zunla-1) genome sequencing project in 2011, we have found no suitable genetic maps for anchoring the massive genomic scaffolds. Similar difficulties were faced by another parallel genome sequencing project of pepper (CM334)^14^. As a result, Dr Doil Choi’s group adopted parent-independent genotyping based on RIL population sequencing to construct an ultra-high-density map for the pseudo-chromosome building of the CM334 genome^16^. By contrast, we customized an Illumina Infinium array, *viz. Cap*SNP15K, to genotype a large intraspecific F_2_ population and construct an intraspecific genetic map (BB-SNP, data not shown) to build the pseudo-chromosomes of the Zunla-1 genome^13^. Both strategies were found to be efficient, with one tentative advantage of the *Cap*SNP15K being the lower ascertainment bias of the SNPs because the 15,000 SNPs were highly filtered and elaborately chosen from the resequencing reads of 20 diverse pepper lines, simultaneously considering the polymorphisms among three related parental lines (BA3, B702 and YNXML).

With regard to the *Cap*SNP15K array, the overwhelming majority of the 12,720 synthesized bead types in fact produced genotyping signals. However, only 8,199 (~64.46%) SNP loci were ultimately found to be normally scorable among all 1,019 investigated samples after the manual removal of abnormal signals (Table 1). The rate of abortive loci was much higher than that of the tomato array^32^. Two possible reasons were as follows: 1) the high rate of duplicate sequences in the pepper genome^13^ may have led to disordered signals or decreased probe specificity; 2) all of the pepper SNPs in the present study were identified *de novo* based on a newly assembled scaffold genome, whereas most of the tomato SNPs were cross-validated by different research groups in advance. Nevertheless, the thousands of well-validated SNP markers in this study showed relatively high genome coverage (~81.18%) and will substantially enrich the pepper marker repertoire. More importantly, the customized array will undoubtedly facilitate high-throughput genotyping and mapping in pepper.

Among the *Capsicum* species, *C. annuum* and *C. frutescens* exhibit a relatively close evolutionary relationship^1^, therefore, these two species have frequently been used as a parental combination to develop interspecific populations for genetic mapping and QTL analyses in pepper^18^,^33^,^34^. Here, we genotyped a large, interspecific F_2_ population (n = 297) derived from BA3 (*C. annuum*) × YNXML (*C. frutescens*) using the newly developed *Cap*SNP15K array and ultimately obtained a high-density SNP genetic map (BY-SNP) for pepper (Fig. 1). The polymorphic rate (97.32%, 5,672/5,828) between BA3 and YNXML was slightly lower than that between BA3 and B702 (98.70%, 7,829/7932, data not shown), indicating that the SNP pool on the *Cap*SNP15K are still biased although the filtering steps as described in Methods were used. Nevertheless, this map still represented the second interspecific map with thousands of markers in pepper following the recently published FA map, which was differently obtained by genotyping an interspecific RIL population using the GeneChip^®^ technology^35^. Despite the total number of markers on BY-SNP (5,569) being less than that of the FA map (16,167), both the genetic bin number (3,826) and map length (1629 cM) of the former were larger than that of the latter (2,105 and 1,380 cM, respectively), indicating a higher recombination rate in our population.

As a pilot study, we used the BY-SNP map to identify QTLs responsible for fruit orientation in pepper, and one major QTL, namely *Up12.1*, was solely detected on chromosome P12 (Fig. 3). This result was consistent with that of genetic analyses from the present and previous studies, again indicating that the erect/pendant variation of pepper fruit was under single-gene control^27^. More interestingly, the LOD peak SNP (scaffold1796.390960) of *Up12.1* was only ~0.4 Mb apart from the major QTL *FP-12.2*, which was recently identified in an intraspecific RIL population of *C. annuum*^16^. This result showed *Up12.1* could be the same QTL as *FP-12.2* despite the population type being different. Furthermore, the different population types may also explain the different number of detected QTLs between ours and recent studies^16^. Nevertheless, the aforementioned results suggested that the interspecific BY-SNP map developed in this study would serve as the basis for future QTL analysis, marker-assisted introgression and comparative mapping in pepper.

In flowering plants, fruit orientation is fundamentally determined by the adaxial-abaxial polarity of the lateral organ pedicel. Furthermore, a number of underlying molecular regulators governing alterations to pedicel orientation have been uncovered, including Arabidopsis *LEAFY*^36^, *KNAT1*/*BP*^37^,^38^, *KNAT2*^39^, *KNAT6*^39^, *CRM1*/*BIG*^40^, *AS1*^41^, *AS2*^42^ and *ATH1*^43^; tomato *SlAGO7*^44^; and tobacco *NtSVP*^45^. However, none of their pepper homologues (Supplementary Table S8) was located within the *Up12.1* region. This result implied that the pepper *up* locus is possibly a new member of the pedicel orientation regulation pathway. The distinct shoot/inflorescence architecture of pepper, which has a sympodial shoot structure with solitary flower^46^, also suggested a possible diversification of the molecular mechanism involved in controlling this plant trait. Meanwhile, among the 65 candidates, *Capana12g000943* near the LOD peak was found to be homologous to the Arabidopsis *purine permease 3* (*PUP3*). *PUP3* is a member of a family of proteins related to *PUP1*, a purine transporter^47^, and may be involved in transporting of purine and purine derivatives such as cytokinin across the plasma membrane. We hypothesized that cytokinin may be another important player in pepper pedicel development in addition to the auxin^48^, and such a hypothesis should be studied further in the future.

Genetic diversity assessment can provide a valuable reference for the effective conservation and utilization of germplasm in crop improvement programmes. Characterization of pepper germplasm diversity, with a geographic origin in China, is rarely reported. Here, we successfully evaluated the genetic relatedness of a moderately large set of diverse pepper lines from the main pepper-producing provinces of China using thousands of newly developed SNP markers. Results showed that genetic diversity level of this panel was relatively low (Table 4), implying that more attentions should be paid to the conservation and broadening of pepper germplasm diversity in future. On the other hand, clustering results based on genetic distance seemed to be associated with morphological classification such as fruit orientation. For example, 45 out of 148 (97.97%) lines with erect fruit were assigned to group II-5 (Fig. 4). In addition, clustering results were supported by population structure analysis and PCA, wherein two population stratifications (P1 and P2) were identified within the 398 *C. annuum* lines (Fig. 5, Supplementary Table S6). All these information laid a foundation for dissecting the genetic architectures of important agronomic traits via association analysis in future.

## Materials and Methods

### SNP array development

SNP calling was performed by mapping the resequencing reads of two parental lines, BA3 (28.5× sequencing depth) and B702 (30.30×), to the Zunla-1 reference genome according to a previous study^13^. To control costs and minimize the impact of SNP ascertainment bias on downstream applications, the following aspects were taken into account when choosing SNPs for microarray design: 1) Priority was given to non-A/T and non-C/G allele variations because of their lower costs compared with other types. 2) Priority was given to retaining the SNPs that show polymorphisms between the two parental combinations, i.e., BA3 vs. B702 and BA3 vs. YNXML. 3) SNPs that show polymorphisms (minor allele frequency >0.25) among the panel of 20 re-sequenced pepper lines representing different horticultural characters were kept^13^. 4) SNPs located in larger scaffolds were preferred, and only 2-3 SNPs were retained in most single scaffolds. In addition, to follow Illumina Infinium assay design quality requirements (Illumina Inc., San Diego, CA, USA), assay design scores were generated for the remaining SNPs. Only SNPs that met the selection criteria for Infinium II assays (one bead type per assay) were retained, and higher assay design scores were preferred over lower scores.

### SNP genotyping

SNP genotyping experiments, including DNA amplification, fragmentation, precipitation, re-suspension, and hybridization, and BeadChip extension, staining and scanning were performed based on the Illumina genotyping platforms in BGI-Shenzhen (Shenzhen, China) according to instructions from the Illumina company (http://www.illumina.com.cn/). The remaining set of SNP markers was analysed using GenomeStudio Genotyping software (v2011, Illumina, Inc.). Because of the large proportion of duplicated sequences and the complex genomic background in the pepper genome, several markers could not be correctly and automatically interpreted by GenomeStudio. To ensure the best cluster, the genotyping signals of each SNP locus of the mapping population and diversity panel lines were clustered and manually adjusted separately. To perform preliminary testing of the genotyping quality of the newly developed array, a set of 6 DNA samples from the parental lines (BA3 and B702) and their hybrids were used to investigate duplicate reproducibility and parent-parent-child (P-P-C) heritability.

### Linkage mapping materials and phenotyping

The female parent, BA3 (*C. annuum*), is a cytoplasmic male sterility (CMS) line. Its maintainer line (near-isogenic line) shows mid-size pendant fruit. By contrast, the pollen donor, YNXML (*C. frutescens*), is an erect type with small fruit. An interspecific F_2_ genetic mapping population including 297 individuals was derived from crossing BA3 and YNXML. Of these, 142 and 155 individuals, together with the parental lines and their hybrid population, were grown successively at the Zengcheng Experimental Station, South China Agricultural University (SCAU), Guangzhou, China (23°N, 113°E), in the autumn of 2011 and 2012. The sub-population from 2012 was previously utilized to identify QTLs for the number of leaves on the primary axis (Nle) trait based on InDel and SSR markers^18^. DNA samples were extracted from young leaves of all plants using the CTAB method and subsequently used for array hybridization. Because of sterility that probably resulted from either interspecific crossing or CMS, only 177 out of 297 (52.57%) individuals could flower and bear fruit successfully. To identify the phenotypes of the segregation population, two kinds of classification methods were used to record the variations in fruit orientation: EP classification divided the individuals into erect (E) and pendant (P) types, whereas another ELV classification classified them as erect (E), lateral pendant (LP) or vertical pendant (VP). Moreover, a minimum of 4 flowers/fruits on each plant were recorded.

### Genetic map construction

A total of 5,828 SNPs were scorable among the parental samples and 297 F_2_ progenies, of which, 156 SNPs showing incorrect or missing genotypes in the parental lines and F_1_ plants were removed. Joinmap4.0^49^ was used for grouping and primary sorting of all remaining markers. The order of markers was optimized as follows: 1) 10% of the total markers with uniform distributions on the Zunla-1 scaffold assembly were chosen to construct an initial framework map. 2) Regression mapping algorithm was used to order as many of the remaining markers as possible to form a new framework map. 3) Maximum likelihood mapping with stricter criteria (Chain length = 10,000; Initial acceptance probability = 0.35; Stop after # chains without improvement = 5,000; Length of burn-in chain = 5,000; Chain length per Monte Carlo EM cycle = 1,500) was applied to integrate the unmapped markers using the new framework map as a fixed order. Recombination values were converted to genetic distances using the Kosambi mapping function. A segregation distortion region (SDR) was defined as a region with five or more adjacent markers with a skewed segregation ratio that differed from the expected ratio (*P*<0.01) according to a previous study^19^.

### QTL analysis

QTL analysis was performed using MapQTL6.0 software^50^ based on the framework map, which included 3,826 bin markers, of the newly constructed genetic map. Interval mapping (IM) method was applied, and the significance threshold of the LOD score was tested by 2,000 permutations and the resultant LOD thresholds for EP and ELV-derived datasets were 5.30 and 5.10, respectively, corresponding to a genome-wide threshold with a *P*-value of 0.01. A genetic confidence interval (CI) was defined as 1-LOD drop from a LOD peak supported regions, and was translated into a physical confidence interval (physical-CI) by projecting markers onto the Zunla-1 reference genome (http://peppersequence.genomics.cn/page/species/index.jsp).

### Diversity analysis materials

A panel of 399 diverse pepper elite/landrace lines, including the two aforementioned parents (BA3 and YNXML), were used for diversity assessment and population structure analysis. Except for the YNXML (*C. frutescens*), all lines are belong to *C. annuum* species (Supplementary Table S6). These lines exhibited a wide range of variability in terms of fruit orientation, pungency, colour, size and other traits. The lines were bred and/or collected in different provinces of China, mainly Guizhou, Guangdong, Yunnan, Hunan, Sichuan, as well as others. All of the seeds were conserved and provided by the Pepper Institute, Zunyi Academy of Agricultural Sciences, Zunyi, Guizhou, China, and the Department of Vegetable Genetics and Breeding, College of Horticulture, South China Agricultural University, Guangzhou, China. After germination in the greenhouse, young leaves from seedlings were used for DNA preparation as described above.

### Genetic diversity analysis

A total of 8,003 scorable SNPs were identified among the panel of 399 diverse lines after removing low-quality data (Table 1). Of these, 574 SNPs representing monomorphisms were removed before further analysis. Then, a final set of 5,149 SNPs was retained using the relatively stringent criteria of locus heterozygosity ≤0.3, missing rate <0.2 and minor allele frequency (MAF) ≥0.05 (Supplementary Table S1). The summary statistics for the genetic diversity of the panel were calculated using Powermarker 3.25 software^51^. The same software was utilized to construct an unweighted pair group method with arithmetic averages (UPGMA) tree based on Nei1983’s genetic distance.

### Genetic structure analysis

The admixture model-based software STRUCTURE V2.3.4^52^ was used to estimate population structure. Because the admixture model is not designed to deal with tightly linked markers, a set of 833 SNPs with an even distribution and relatively large interval (~3.2 Mb) across the Zunla-1 genome (Supplementary Table S1) were selected to estimate the population structure of the 398 *C. annuum* lines with the exception for YNXML. The tested K was set from 1 to 10 and analyses were repeated five times with 100,000 Markov Chain Monte Carlo (MCMC) replicates and 100,000 burn-ins. ΔK^53^ was used to determine the optimal K for population structure. Principal component analysis of 398 *C. annuum* lines based on 6470 SNPs with MAF ≥0.05 was performed using the software EIGENSOFT^54^ in this study.

## Additional Information

**How to cite this article**: Cheng, J. *et al*. Development of a SNP array and its application to genetic mapping and diversity assessment in pepper (*Capsicum* spp.). *Sci. Rep.* **6**, 33293; doi: 10.1038/srep33293 (2016).

## Supplementary Material

Supplementary File S1

Supplementary Table S1

Supplementary Table S4

Supplementary Table S6

## Figures and Tables

**Table 1:** Utilization and efficiency of pepper *Cap*SNP15K array among 1,019 investigated samples.

Materials	Number	Scorable		Located scaffold		Percentage (%)^b^
		Count	Percentage (%)^a^	Count	Length (bp)	
Quality control	6	8,003	62.92	5,013	2,688,775,059	80.28
Elite/landrace lines	399	8,003	62.92	5,013	2,688,775,059	80.28
BA3 × YNXML F_2_	297	5,828	45.82	3,682	2,451,521,991	73.19
BA3 × B702 F_2_^c^	317	7,932	62.36	5,001	2,671,564,846	79.76
All investigated	1,019	8,199	64.46	5,107	2,719,081,414	81.18

^a^The ratio of scorable loci to successfully synthesized 12,720 loci.

^b^The ratio of located scaffold length to that of Zunla-1 genome (3,349,397,670 bp).

^c^The detailed information will be published elsewhere.

**Table 2:** Statistical results of the high-density interspecific SNP map of pepper.

LG	Chr.	# Marker	# Bin	Bin interval (cM)		Genetic length (cM)	Physical length (Mb)^a^	Distorted marker^b^	SDRs
				Maximum	Average				
1	P1, P8	909	662	6.88	0.42	277.66	381.85^c^	655	8
2	P2	328	255	1.93	0.43	109.43	161.58	41	1
3	P3	460	327	1.89	0.41	133.18	260.71	396	8
4	P4	400	294	3.34	0.47	137.68	214.27	53	3
5	P5	394	284	13.83	0.48	134.76	217.05	71	5
6	P6	332	244	1.94	0.47	113.52	219.01	13	0
7	P7	866	455	2.27	0.31	141.2	219.37	32	0
8	P1	98	79	3.21	0.7	54.68	44.76	46	2
9	P9	458	299	1.81	0.36	108.51	238.18	84	3
10	P10	315	227	8.79	0.51	114.86	205.7	99	6
11	P11	577	393	3.19	0.39	150.95	220.29	15	0
12	P12	432	307	6.04	0.5	152.4	229.85	167	3
Mean	—	464.08	318.83	—	0.45	135.74	202.8	139.33	3.25
Total	—	5,569	3,826	—	—	1,628.83	2230.77	1,672	39

^a^This length included the 1000 N between the adjacent scaffolds.

^b^Based on the 4879 SNPs with missing rate less than 10% among the 297 F_2_ progenies.

^c^Equal to the sum of P1 length (228.91 Mb) and P8 length (152.94 Mb).

**Table 3:** QTL results for fruit orientation based on two classification methods.

QTL	Method	LG	LOD	Genetic interval (cM)^a^				Physical interval (Mb)				R^2^ (%)	Additive	Dominance
				Left	Peak	Right	Size	Left	Peak	Right	Size			
*Up12.1*	EP	12	40.11	35.79	35.97	39.32	3.52	36.54	37.35	41.06	4.52	64.80	−0.91	−0.71
	ELV	12	28.19									52.00	−0.67	−0.30

^a^1-LOD drop confidence interval, the LOD peak markers of *Up12.1* was scaffold1796.390960.

**Table 4:** The summary statistics for genetic diversity among the pepper lines.

Group	Sample size	MAF^a^	Genotype number	Allele number	Gene diversity^b^	Heterozygosity	PIC^c^
I	101	0.21	2.89	1.98	0.29	0.08	0.24
II	296	0.25	2.99	2.00	0.34	0.20	0.27
II-1	3	0.17	1.58	1.51	0.21	0.13	0.16
II-2	11	0.17	2.13	1.79	0.24	0.06	0.19
II-3	1	0.01	1.00	1.01	0.00	0.01	0.00
II-4	25	0.11	1.93	1.76	0.17	0.01	0.14
II-5	256	0.24	2.99	2.00	0.33	0.23	0.26
Total	399	0.26	3.00	2.00	0.36	0.17	0.29

^a^MAF, minor allele frequency.

^b^The gene diversity of groups II-1 and II-3 were calculated based on 5,142 and 5,062 SNPs, respectively.

^c^PIC, polymorphism information content.
