# Supplementary material for: Development of a SNP array and its application to genetic mapping and diversity assessment in pepper (Capsicum spp.)
Source: Sci Rep. 2016 Sep 13;6:33293. doi: 10.1038/srep33293 (PMC5020730; doi:10.1038/srep33293)
Supplement: Supplementary File S1 [file srep33293-s1.pdf]

Development of a SNP array and its application to genetic mapping and diversity assessment in pepper (*Capsicum* spp.)

Jiaowen Cheng[1¶], Cheng Qin[2, 3¶], Xin Tang[1¶], Huangkai Zhou[4], Yafei Hu[5], Zicheng Zhao[6], Junjie Cui[1], Bo Li[1], Zhiming Wu[7], Jiping Yu[2]*, Kailin Hu[1]*

Supporting Information Legends

Supplementary Figure S1. The frequency distribution of 8,199 scorable SNPs with various types and locations.

A, Type; **B**, location.

Supplementary Figure S2. Phenotypes and schematic diagrams of fruit orientation in pepper.

A, The erect phenotype, with a vertical upward pedicel and fruit tip; **B**, the lateral pendant phenotype, with a near-horizontally orientated pedicel and fruit tip; **C**, the vertical pendant phenotype, with a straight-down pedicel and fruit tip.

Supplementary Table S1. The information of 8,199 scorable SNP loci on the *Cap*SNP15K array of pepper.

Supplementary Table S2. Estimation of duplicate reproducibility based on 8,003 SNPs.

Supplementary Table S3. Estimation of P-P-C heritability based on 8,003 SNPs.

Supplementary Table S4. The genetic and physical positions of 5,672 SNPs used for genetic mapping.

Supplementary Table S5. List of 65 candidate genes located in 1-LOD drop CI for *up* locus of pepper.

Supplementary Table S6. The horticultural characterization, grouping and population structure of the 399 pepper elite/landrace lines.

Supplementary Table S7. Comprison of 398 *C. annuum* lines between clustering and structure analysis.

Supplementary Table S8. List of genes implicated in the regulation of pedicel orientation and their closest homologues in pepper.

Supplementary Tables

Supplementary Tables S1 is provided in the form of a single file.

Supplementary Table S2. Estimation of duplicate reproducibility based on 8,003 SNPs.

Sample	Repeat 1	Repeat 2	# Correct	# Error	Total[a]	Duplicate reproducibility(%)
BA3	BA3-1	BA3-2	7,782	0	7,782	100.00
B702	B702-1	B702-2	7,929	0	7,929	100.00

[a] The number of SNPs that presented in two repeats.

Supplementary Table S3. Estimation of P-P-C heritability based on 8,003 SNPs.

Child-DNA	Parent 1	Parent 2	# Correct	# Error	Total[a]	P-P-C heritability (%)
F1-BB-1	BA3-1	B702-1	4,980	204	5,184	96.06
F1-BB-2	BA3-2	B702-2	4,638	328	4,966	93.40

[a] The number of SNPs that presented in three parental and F_1 samples.

Supplementary Tables S4 is provided in the form of a single file.

Supplementary Table S5. List of 65 candidate genes located in 1-LOD drop CI for *up* locus of pepper.

Gene name	Start position	End position	Gene symbol	Homologous species	Homologous protein
Capana12g000941	36,558,550	36,559,306	PFK1	*Arabidopsis thaliana*	6-phosphofructokinase 1
Capana12g000942	36,617,084	36,617,572	New gene		
Capana12g000943	36,655,866	36,659,865	PUP3	*Arabidopsis thaliana*	Purine permease 3
Capana12g000944	36,678,874	36,679,269	New gene		
Capana12g000945	36,962,779	36,964,097	New gene		
Capana12g000946	36,964,963	36,966,866	New gene		
Capana12g000948	37,018,517	37,030,992	New gene		
Capana12g000949	37,036,408	37,037,557	New gene		
Capana12g000950	37,317,274	37,317,706	New gene		
Capana12g000951	37,319,249	37,320,811	New gene		
Capana12g000952	37,376,725	37,380,297	New gene		
Capana12g000954	37,459,071	37,460,454	New gene		
Capana12g000955	37,628,063	37,628,443	New gene		
Capana12g000958	37,644,791	37,650,731	DRG2	*Arabidopsis thaliana*	Developmentally regulated G-protein 2
Capana12g000959	37,683,574	37,688,329	New gene		

Capana12g000960	37,689,586	37,689,918	EFL3	*Arabidopsis thaliana*	Protein ELF4-LIKE 3
Capana12g000962	37,733,635	37,734,657	New gene		
Capana12g000964	37,739,818	37,740,560	New gene		
Capana12g000965	37,783,070	37,785,471	New gene		
Capana12g000966	37,786,745	37,787,077	EFL3	*Arabidopsis thaliana*	Protein ELF4-LIKE 3
Capana12g000967	37,891,276	37,893,142	MYBC	*Zea mays*	Anthocyanin regulatory C1 protein
Capana12g000968	37,915,594	37,916,120	New gene		
Capana12g000969	37,928,500	37,931,398	ATG12	*Medicago truncatula*	Ubiquitin-like protein ATG12
Capana12g000970	37,933,621	37,934,247	ABP19A	*Prunus persica*	Auxin-binding protein ABP19a
Capana12g000971	38,186,615	38,187,046	New gene		
Capana12g000972	38,264,977	38,265,832	At5g01020	*Arabidopsis thaliana*	Serine/threonine-protein kinase At5g01020
Capana12g000973	38,358,798	38,359,109	New gene		
Capana12g000975	38,359,822	38,360,898	NAD5	*Triticum aestivum*	NADH-ubiquinone oxidoreductase chain 5
Capana12g000976	38,430,680	38,433,697	At1g72540	*Arabidopsis thaliana*	Putative receptor-like protein kinase At1g72540
Capana12g000978	38,980,737	38,981,694	New gene		
Capana12g000979	39,002,580	39,003,837	New gene		
Capana12g000980	39,016,908	39,019,920	New gene		

Capana12g000981	39,019,948	39,021,887	New gene		
Capana12g000982	39,024,682	39,027,683	New gene		
Capana12g000983	39,045,217	39,049,182	SEC22	*Arabidopsis thaliana*	25.3 kDa vesicle transport protein
Capana12g000984	39,049,950	39,051,757	New gene		
Capana12g000985	39,052,779	39,055,821	New gene		
Capana12g000986	39,056,366	39,056,899	New gene		
Capana12g000987	39,064,202	39,082,032	Nup107	*Mus musculus*	Nuclear pore complex protein Nup107
Capana12g000990	39,269,034	39,274,278	TIFY10B	*Arabidopsis thaliana*	Protein TIFY 10B
Capana12g000991	39,290,108	39,297,758	Tmem87a	*Xenopus tropicalis*	Transmembrane protein 87A
Capana12g000992	39,314,704	39,316,697	CBSDUF7	*Arabidopsis thaliana*	DUF21 domain-containing protein At1g47330
Capana12g000993	39,321,504	39,322,085	New gene		
Capana12g000994	39,465,598	39,467,865	New gene		
Capana12g000996	39,767,987	39,776,493	Fig4	*Mus musculus*	Polyphosphoinositide phosphatase
Capana12g000999	39,881,247	39,889,683	GLIP5	*Arabidopsis thaliana*	GDSL esterase/lipase 5
Capana12g001002	39,992,532	39,993,545	New gene		
Capana12g001003	39,995,458	39,996,613	New gene		
Capana12g001004	40,021,604	40,022,422	New gene		

Capana12g001005	40,100,125	40,112,227	New gene		
Capana12g001007	40,472,266	40,473,107	ATP5E	*Ipomoea batatas*	ATP synthase subunit 5
Capana12g001008	40,474,850	40,475,953	New gene		
Capana12g001009	40,611,845	40,613,824	Exoc7	*Rattus norvegicus*	Exocyst complex component 7
Capana12g001010	40,615,725	40,618,088	New gene		
Capana12g001011	40,622,428	40,636,788	At4g18375	*Arabidopsis thaliana*	KH domain-containing protein At4g18375
Capana12g001012	40,869,247	40,883,486	SWI3D	*Arabidopsis thaliana*	SWI/SNF complex subunit SWI3D
Capana12g001013	40,885,348	40,890,485	RABG3F	*Arabidopsis thaliana*	Ras-related protein RABG3f
Capana12g001014	40,893,131	40,896,956	PERK4	*Arabidopsis thaliana*	Proline-rich receptor-like protein kinase PERK4
Capana12g001015	40,946,680	40,960,886	At4g34450	*Arabidopsis thaliana*	Coatomer subunit 3
Capana12g001016	40,961,437	40,962,249	New gene		
Capana12g001017	40,963,010	40,971,221	GBB2	*Nicotiana tabacum*	Guanine nucleotide-binding protein subunit beta-2
Capana12g001018	40,974,606	40,981,738	At4g34480	*Arabidopsis thaliana*	Glucan endo-1,3-beta-glucosidase 7
Capana12g001019	40,986,390	40,996,534	cap	*Dictyostelium discoideum*	Adenylyl cyclase-associated protein
Capana12g001020	41,000,982	41,005,216	At4g34500	*Arabidopsis thaliana*	Probable receptor-like serine/threonine-protein kinase At4g34500
Capana12g001021	41,033,062	41,033,647	New gene		

Supplementary Tables S6 is provided in the form of a single file.

Supplementary Table S7. Comprison of 398 *C. annuum* lines between clustering and structure analysis.

	I	II	II-1	II-2	II-3	II-4	II-5	Other[a]	Total
P1	89	10	2	4	0	0	4	0	99
P2	12	286	1	7	1	25	252	1	299
Total	101	296	3	11	1	25	256	1	398

[a]Refer to the line Y126.

Supplementary Table S8. List of genes implicated in the regulation of pedicel orientation and their homologues in pepper.

Gene symbol	Species	Accession number	Protein description	Zunla-1 closest homologue
LFY	Arabidopsis	NP_200993.1_1	transcription factor	*Capana03g000623*
KNAT1/*BP*	Arabidopsis	NP_192555.1	homeobox protein knotted-1-like 1	*Capana04g000755*
KNAT2	Arabidopsis	NP_177208.2	homeobox protein knotted-1-like 2	*Capana11g001260*
KNAT6	Arabidopsis	NP_850951.2	homeobox protein knotted-1-like 6	*Capana11g001260*
CRM1/*BIG*	Arabidopsis	NP_186875.2	auxin transport protein	*Capana02g003223*
AS1	Arabidopsis	NP_181299.1	transcription factor	*Capana09g001823*
AS2	Arabidopsis	NP_001077777.1	transcription factor	*Capana12g002602*
ATH1	Arabidopsis	NP_195024.1	homeobox protein ATH1	*Capana08g000327*
SlAGO7	Tomato	NP_001266209.1	protein argonaute 7	*Capana01g002131*
NtSVP[a]	Tobacco	mRNA_128733_cds; mRNA_131449_cds; mRNA_171558_cds	MADS-box transcription factor	*CaSVP*/*CaJOINTLESS*/*Capana12g002710*

[a]Its accession numbers were from the SOL Genomics Network (https://solgenomics.net/).

Supplementary Figures

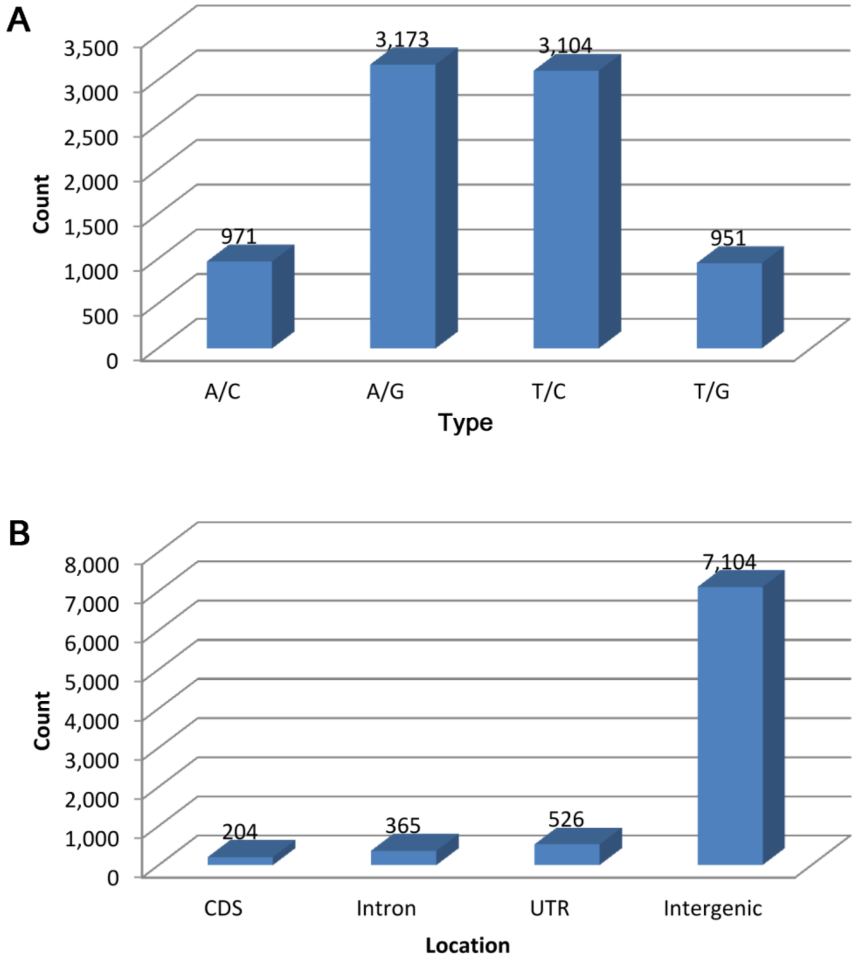

Supplementary Figure S1. The frequency distribution of 8,199 scorable SNPs with various types and locations.

A, Type; **B**, location.

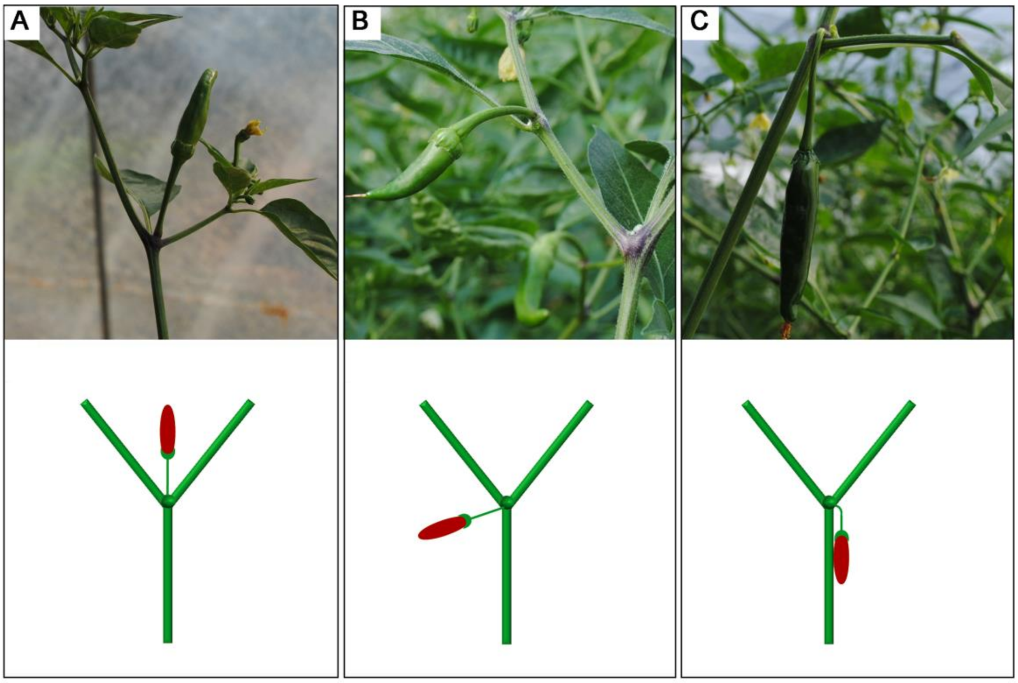

Supplementary Figure S2. Phenotypes and schematic diagrams of fruit orientation in pepper. **A**, The erect phenotype, with a vertical upward pedicel and fruit tip; **B**, the lateral pendant phenotype, with a near-horizontally orientated pedicel and fruit tip; **C**, the vertical pendant phenotype, with a straight-down pedicel and fruit tip.
